# Impact of fibromyalgia syndrome diagnosis and treatment experiences on health information-seeking behaviour: A cross-sectional online survey

**DOI:** 10.1177/20494637261447443

**Published:** 2026-05-06

**Authors:** Juliette Allen, Andreas Goebel, Nicholas Fallon

**Affiliations:** 1 Department of Psychology, Institute of Population Health, 4591 University of Liverpool, Liverpool, UK; 2 Pain Research Institute, Institute of Life Course and Medical Sciences, 4591 University of Liverpool, Liverpool, UK

**Keywords:** fibromyalgia syndrome, health information, clinical pathway, diagnosis, treatment, chronic pain, online support

## Abstract

**Background:**

Patients with fibromyalgia syndrome (FMS) often report prolonged diagnostic pathways and inadequate care, prompting reliance on self-management and online health information. This study aimed to quantify the association between specific clinical experiences and the sources and extent of health information-seeking behaviours in people with FMS.

**Methods:**

A cross-sectional online survey was completed by adults who self-reported a diagnosis of FMS. Measures assessed symptom severity, diagnostic and treatment experiences, frequency and duration of symptom flares, perceived stigma and caring from healthcare professionals, and engagement with health information sources, including traditional and digital platforms. Associations between clinical experiences and health information-seeking behaviours were examined using non-parametric tests and hierarchical regression analyses.

**Results:**

A total of 384 adults completed the survey (75.3% female; median age 41 years). Most participants reported experiencing symptom flares (88.0%), occurring approximately every 2–3 weeks and lasting a median of 3 days. Participants reported significantly more negative than neutral experiences across multiple diagnostic and treatment variables, including diagnostic difficulty and challenges accessing specialist care. Most respondents (84.1%) actively sought health information, most commonly from healthcare professionals, websites, and online network platforms. Nearly half reported difficulties accessing satisfactory health information. Greater diagnostic difficulty, difficulty finding a specialist, and higher perceived caring from healthcare professionals independently predicted engagement with a wider range of health information sources.

**Conclusions:**

Patients with FMS frequently report dissatisfaction with their clinical experiences. Positive and negative diagnostic and treatment experiences are associated with the extent of health information-seeking among people with FMS. These findings highlight the importance of clinical experiences in shaping how patients seek health information and underscore the role of supportive clinical relationships in fostering informed and collaborative care.

## Introduction

Fibromyalgia syndrome (FMS) is a polysymptomatic chronic pain condition characterised by symptoms of widespread musculoskeletal pain, fatigue, stiffness, cognitive dysfunction, sleep and autonomic disturbances, regional pain syndromes, and hypersensitivity to external stimuli.^1^ There is a 2% prevalence of FMS as a single diagnosis,^2^ climbing to 30% as a dual diagnosis alongside autoimmune rheumatological conditions.^3^ The impact of FMS can be severe, with one-third of patients disabled by their symptoms.^4^ FMS predominantly impacts women, accounting for 80–90% of cases,^5^ and women with FMS also experience a greater range of pain and other symptoms.^6^ FMS is a complex condition with an unknown aetiology, but proposed mechanisms include dysregulated pain processing and peripheral nerve abnormalities,^7,8^ and recent evidence suggests an important role for autoimmune factors.^9^

People with FMS often face diagnostic delays and inconsistent care; in the United Kingdom (UK), the diagnostic pathway is variable, with reports indicating that a confirmed diagnosis may take approximately 7 years.^10,11^ These delays have been attributed to overlapping clinical features with commonly comorbid conditions, the absence of objective diagnostic tests, lack of integrated care pathways, and symptom heterogeneity with fluctuating periods of symptom exacerbation.^12–14^ Approximately three-quarters of FMS patients report the experience of ‘flares’, a period of exacerbated symptoms which may last from a few days, up to 20 weeks.^15^ Flare episodes may further complicate diagnosis and treatment, yet their epidemiology and characteristics remain greatly under-researched.^15,16^ Studies have shown that delays in diagnosis contribute to patient frustration^13^ and a greater likelihood of misdiagnoses.^14^

Many FMS patients report dissatisfaction with the support received from healthcare services,^17^ and consequently adopt self-management strategies, which may become unsustainable during periods of severe symptoms.^18^ In this context, FMS patients draw on a range of health information and support sources, including both traditional sources (such as pharmacy leaflets and support groups) and digital resources (peer networks and health websites/forums).^19,20^ Digital resources are a growing and accessible route to emotional support, social contact, and advice regarding symptoms and stigmatisation.^21^ Online networks are also a source of knowledge sharing and education for healthcare-related decision-making.^22,23^ However, the quality, completeness, and accessibility of health information regarding FMS have been evaluated as poor.^23^ Additionally, it has been posited that a cyclical relationship exists between chronic pain, health anxiety, and the search for online health information.^24^

While a growing body of research has explored why people with FMS seek health information online, less is known about how specific clinical experiences shape these behaviours. A recent qualitative study found that uncertainty, fear, and worry often motivate individuals with FMS to engage with digital resources, particularly when faced with prolonged diagnostic processes.^25^ Similarly, a qualitative synthesis reported that patients often feel dependent on online resources due to insufficient information from healthcare professionals.^26^ These findings suggest that experiences across diagnostic and treatment pathways, hereafter referred to as ‘clinical experiences’, may play an important role in shaping health information-seeking behaviour. However, quantitative research examining the relationship between specific clinical experiences and information-seeking remains limited.

Therefore, the aim of this study was to investigate and quantify the relationship between patients’ clinical experiences and health information-seeking behaviours using a cross-sectional online survey. Specifically, this study aimed to examine how experiences related to diagnosis and treatment were associated with the extent and sources of health information-seeking, such as online networks, health websites, and direct contact with healthcare professionals. We hypothesised that there would be an association between negative clinical experiences and greater health information-seeking among FMS patients.

## Methods

### Design

This study employed a cross-sectional design, administering online surveys between January and April 2022 among FMS patients in the UK. A cross-sectional survey design was intentionally selected to enable the identification of patterns and health-information-seeking behaviour at scale in an under-researched population, consistent with established methodological approaches in chronic pain research.^10,27–31^ Ethical approval was received from the University of Liverpool Health and Life Science Research Ethics Committee (10771).

Participants were recruited through voluntary and opportunity sampling. Research advertisements were shared across patient support groups and FMS information pages on Facebook and Instagram. Additionally, patients who were already known to the research team and had consented to be contacted for further studies were sent a link to the survey. The Fibromyalgia UK Charity also promoted the study on their research page.

### Participants

Participants were eligible for inclusion if they self-reported a diagnosis of FMS. Participants who were not fluent in English were excluded from the study. Patients with dual diagnoses were included due to the previously reported high co-morbidity prevalence between FMS and other medical/psychiatric conditions.^32^

All participants provided informed online consent at the start of the survey for their data to be used for research purposes.

### Materials

Participants were asked to report demographic characteristics: age, sex, ethnicity, highest level of education completed, and employment status. Subsequently, a survey was administered through Qualtrics^33^ and Gorilla,^34^ covering the following themes: FMS symptoms, diagnostic and treatment experiences, perceived understanding of FMS, and health information-seeking. Participants were able to leave the survey and return at a later time if fatigued. Upon completion, participants were able to anonymously provide their details to be entered in a prize draw.

### Survey development

To develop the survey, a review of the literature identified critical gaps and inconsistencies in FMS patients’ diagnostic and treatment experiences. It also highlighted themes seen as significant challenges for FMS patients, such as flare-ups, fatigue, and health information availability. This initial review served as a foundation for outlining the preliminary content and themes to be addressed in the survey. Survey designs from several patient surveys were also reviewed to ensure methodological rigour.^35–43^ These studies outlined a common approach to designing survey items based on patient experiences; they also assessed diagnostic pathways, patient experiences, and treatment outcomes, offering a structured framework for the current survey design. This framework was subsequently tailored to reflect the distinctive experiences of FMS patients, modifying questions to better align with their specific symptoms and challenges. The research team conducted an iterative review process to ensure that each question was directly aligned with the study’s research objectives: redundancies were removed to maintain a clear focus on critical aspects of FMS experience; language and phrasing were adjusted for readability. The complete 33-item survey is available in Supplemental Materials (S1).

To explore the theme of FMS symptoms, questions regarding the range, frequency, and severity of symptoms were included. Questions explored current treatments being used and participants’ experiences of flares. An example of these questions includes ‘In the past week, how severe has the *chronic widespread pain* you experience from your FMS been on average?’; this was measured on an 11-point scale where 0 = ‘no pain’ and 10 = ‘maximum pain’. Another example includes ‘How often do you experience FMS flares?’.

To explore diagnostic and treatment experiences, eleven questions regarding the time taken to receive a diagnosis, medical doctors (MDs) involved in the diagnostic pathway, and additional clinical diagnoses were included. Examples of questions in this theme include ‘How many medical doctors did you see about your widespread pain until you received your diagnosis of FMS?’. Another example includes ‘Overall, how easy/difficult was your experience in receiving an FMS diagnosis?’ measured on a 5-point scale from 1 = very easy, to 3 = neither easy nor difficult, and 5 = very difficult.

To explore the theme of perceived understanding of FMS, six questions were administered. The term ‘understanding’ was not defined in order to encompass its multifaceted nature. This term may include various cognitive and emotional dimensions, such as awareness of symptoms, exacerbating factors, and daily life implications. To include the intricate nuances of FMS patient experiences, participants interpreted ‘understanding’ based on their perspectives and lived experiences. Examples of questions in this theme include ‘How well do you feel you understand FMS?’ and ‘How have your family and friends understood your FMS?’, all measured on a 5-point scale from 1 = very poor to 5 = very well.

The final theme, health information-seeking, involved 6 questions regarding sources of health information and motives for health information-seeking. Examples included ‘Which of the following health websites do you use to get your health information from?’ and ‘Select the reasons which best explain why you have searched for information in the last 12 months’. Questions presented a list of options where respondents could select multiple items or alternatively enter ‘other’ information using a free text box.

### Sample size

An a priori power analysis was conducted using G*Power (version 3.1.9.7)^44^ to determine the minimum sample size for hierarchical regression analyses aimed at predicting health information-seeking behaviour based on patients’ clinical experiences, while controlling for sociodemographic factors. The analysis used F-tests for linear multiple regression: fixed model, R^2^ increase, with an effect size of 0.048, derived from Li et al. (2015)^45^; this study explored online health information-seeking with predictors, including socio-demographic factors in block 1 and health-related variables in block 2. The formula f^2^ = ΔR^2^/(1 - R^2^ total) was used, yielding f^2^ = 0.048. A significance level of 0.05 and power of 0.80 were set. Nine predictors were included, four confound variables in block 1 and five predictor variables in block 2 (see data analysis below for details). The analysis yielded a minimum sample size of 273 participants for sufficient power to detect unique contributions from block 2.

### Data analysis

#### Normality testing, distribution, and group differences

Statistical analyses were carried out using SPSS 27.0 software for Windows. Quantitative data were analysed descriptively, summarised by the number of respondents answering a question in each category. All analyses were two-tailed, and probabilities of less than 0.05 were used for significance testing. Respondents were able to skip questions, so variables were analysed with *n* equal to the number of responses to that question. The Shapiro–Wilk test was used due to its well-documented strength and sensitivity in detecting deviations from normality^46^; no variables demonstrated a normal distribution. Therefore, non-parametric tests were used in subsequent analyses; the median (Md) and interquartile range (IQR) are reported unless otherwise stated. Group differences are available in Supplemental Materials (S2).

#### Descriptive analysis of clinical experience variables

To explore whether participants reported positive, neutral, or negative clinical experiences, one-sample Wilcoxon signed-rank tests were conducted. This approach was selected as a non-parametric alternative to the one-sample *t*-test, appropriate for non-normally distributed variables.^47,48^ The test enabled the assessment of whether the median response differed significantly from a theoretically defined neutral midpoint, providing an indication of the overall direction (positive vs negative) of reported experiences rather than descriptive summaries.

Eight clinical experience variables were assessed. Seven were measured using 5-point bimodal scales. The first two variables, *Diagnostic Difficulty* and *Difficulty Finding a Specialist*, used response options ranging from 1 (very easy) to 5 (very difficult). Four variables, *Understanding of FMS*, *Understanding of FMS Symptoms*, *Understanding of FMS from Family and Friends*, and *Understanding of FMS from Medical Doctors*, used response options from 1 (very poorly) to 5 (very well). The seventh variable, *Perceived Level of Caring from Medical Doctors*, was rated from 1 (very uncaring) to 5 (very caring). For these seven items, a test value of 3 was used to represent a theoretically neutral midpoint. This corresponded to responses such as ‘neither easy nor difficult’, ‘neither well nor poorly’, or ‘neither caring nor uncaring’. The eighth variable, *Perceived Negative Judgement from Medical Doctors*, was measured using a 4-point frequency scale ranging from 1 (never) to 4 (always). For this variable, a test value of 1 was used to represent the neutral reference point, indicating no experience of negative judgement. The effect size (*r*) was calculated by dividing the *Z* statistic by the square root of *n*.

#### Hierarchical regressions for health information-seeking variables

To investigate health information-seeking, three hierarchical regressions were conducted to examine whether patients’ individual clinical experiences predicted (a) the total number of sources used for health information-seeking, (b) the total number of social media sites used for health information-seeking, and (c) the total number of health sites used for health information-seeking.

Age, Sex, Education, and Employment were entered as covariates in the first step; Diagnostic Difficulty, Difficulty in Finding a Specialist, Perceived Understanding of FMS from MDs, Perceived Level of Caring from MDs, and Perceived Negative Judgment from MDs were input in step 2 as predictors. These variables were selected as they capture experiences of the FMS clinical pathway which were also hypothesised to influence information-seeking. There was independence of residuals for all three models, as assessed by a Durbin–Watson statistic of 1.96, 1.85, and 2.00, respectively. There were linearity and homoscedasticity across all models, as assessed by partial regression plots and plots of studentized residuals against the predicted values. There was no evidence of multicollinearity across any models, as assessed by tolerance values greater than 0.1.

## Results

### Demographics

A total of 384 participants who self-reported a formal diagnosis of FMS completed the survey. This included 289 female (75.3%), 94 male (24.5%) participants, and 1 participant who chose to not report their sex (0.3%). Participants were aged between 19 and 84 years old, with a median age of 41 years (IQR = 21). The most frequently reported ethnicity was White British/White European (97.1%). Patients were most frequently working full-time (39.8%) or were unemployed (37.8%). The current study sample aligns broadly with demographic patterns observed in the UK fibromyalgia population. The age of participants is consistent with reported age distributions in recent FMS survey studies.^49–52^ The current sample showed a female predominance (75%).

### Symptoms, diagnosis, & treatment experiences

**Figure 1.**
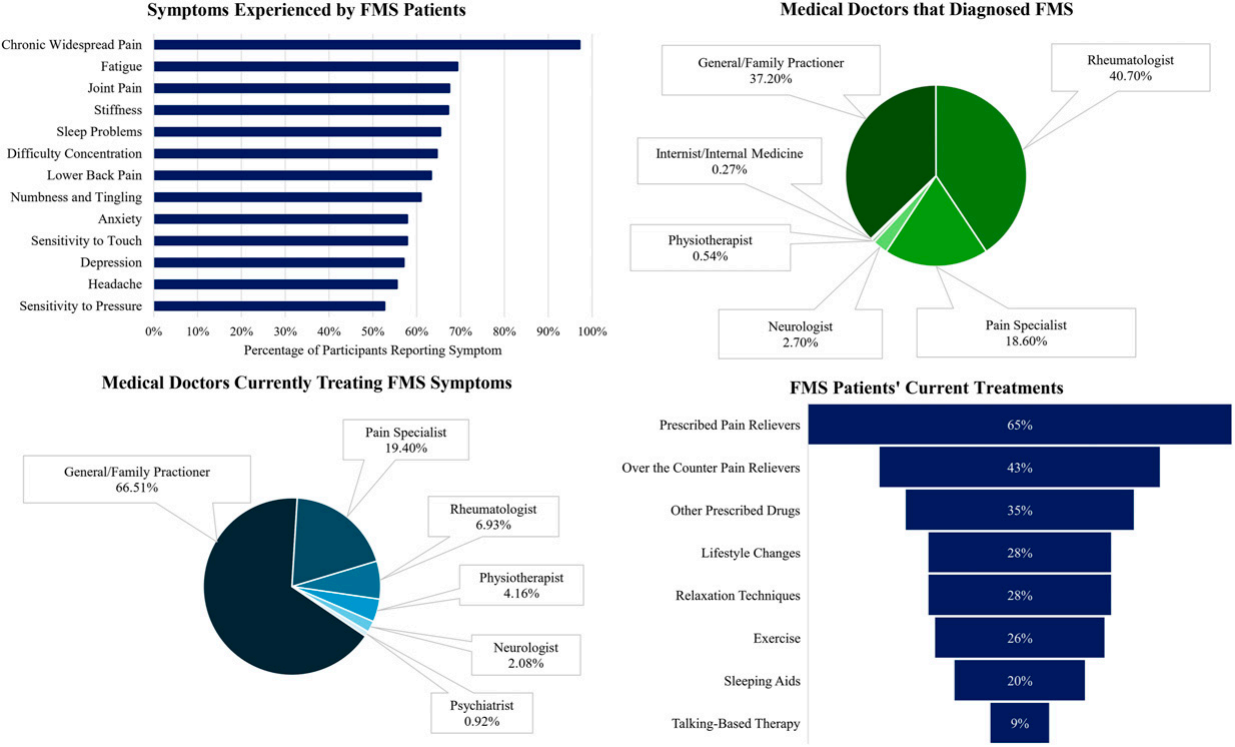
FMS experience and management. The top-left panel displays the symptom profile of FMS patients, illustrating the percentage of patient respondents reporting common symptoms. The top-right panel presents a pie chart depicting the relative proportion of specialist practitioners who most frequently diagnosed FMS. The bottom-left panel illustrates the relative proportion of medical doctors currently treating FMS symptoms. The bottom-right panel depicts the percentage of patient respondents reporting the use of common treatment approaches.

The most common symptoms reported by patients were *Chronic Widespread Pain* (*CWP*) (97.1%), *Fatigue* (69.3%), and *Joint Pain* (67.4%). Participants self-reported a median of 6 months (IQR = 35.5) to receive a diagnosis after first seeing an MD for their FMS symptoms. Participants were diagnosed with FMS by *Rheumatologists* (38.3%), *General Practitioners* (35.9%), and *Pain Specialists* (18%). Following diagnosis, patients were most frequently treated by *General Practitioners* (75%), *Pain Specialists* (21.9%), and *Rheumatologists* (7.8%). The most frequently reported treatments were *Prescribed Pain Relief* (64.6%), *Over-the-Counter Pain Relief* (42.7%), or *Other Prescribed Drugs* (35.2%). Only 9.4% of patients reported to have received *Talking Therapies* as a treatment for their FMS symptoms (see Figure 1 for a summary of these findings).

**Figure 2.**
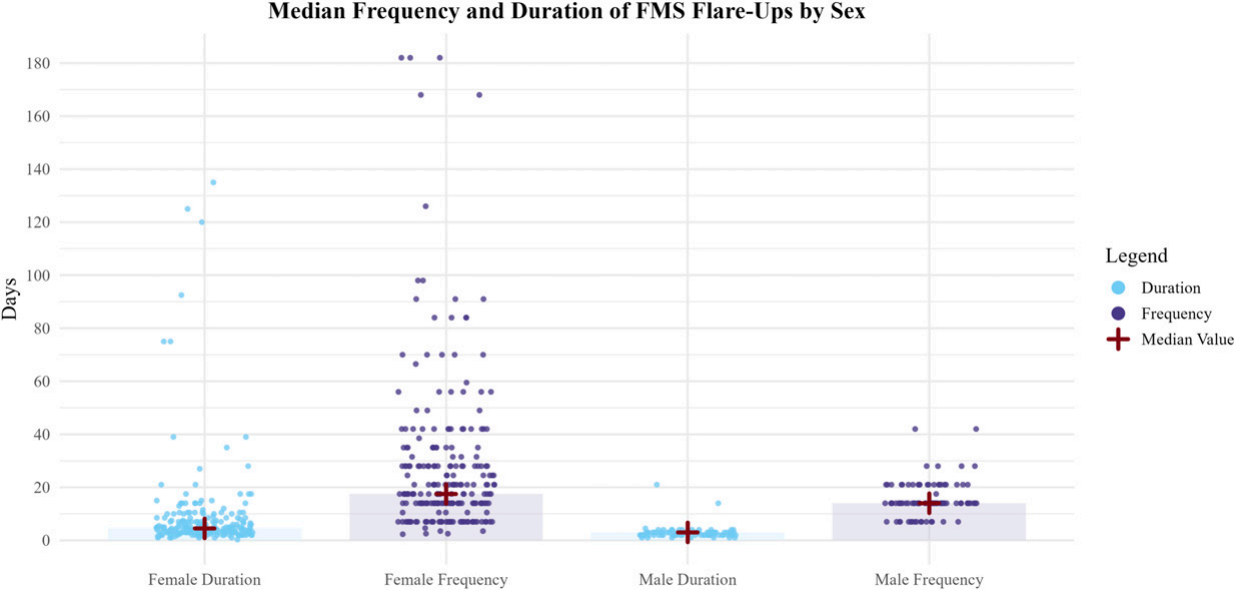
Median frequency and duration of FMS flare-ups by sex. Figure presenting the median number of days for frequency and duration of FMS flare-ups stratified by sex (female and male). Bars show median values with translucent fills differentiating duration and frequency, overlaid with individual data points as coloured circles. The red cross indicates the median for each group. Note that for frequency, higher values represent less frequent flare-ups (i.e. more days between episodes).

With 88.02% of the sample reporting some experience of flares, the median frequency was every 2.5 weeks (IQR = 2); the median duration was 3 days (IQR = 3.63). As demonstrated in Figure 2, male participants reported more frequent experiences of flares (a median of 2 weeks (IQR = 1)) but shorter duration of flares (a median of 3 days (IQR = 1)) compared to female patients (a median of every 2.5 weeks (IQR = 2.5), and a median of 4.5 days (IQR = 4.5), respectively). See Table 1 for descriptive statistics.

**Table 1.** Clinical descriptive statistics.

Variables	Female					Male					Total				
	Md	IQR	M	SD	N	Md	IQR	M	SD	N	Md	IQR	M	SD	N
Age	45	19.50	44.37	12.11	289	29	5	30.68	7.97	94	41	21	41.06	12.71	384
Frequency of FMS flares (weeks)	2.50	2.50	4.05	4.31	232	2.00	1.00	2.23	0.94	92	2.50	2.00	3.55	3.77	325
Duration of FMS flares (days)	4.50	4.50	8.86	17.44	219	3.00	1.00	2.92	2.42	90	3.00	3.63	7.12	14.95	310
Severity of chronic widespread pain	7.00	3.00	6.87	2.14	287	5.00	2.00	5.29	1.67	92	7.00	3.00	6.49	2.14	380
Severity of fatigue	8.00	3.00	7.59	2.04	287	5.00	3.00	5.42	1.83	89	7.00	4.00	7.08	2.19	377
Number of symptoms	12.00	3.00	10.97	3.91	289	1.00	0.00	1.86	2.66	94	11.00	12.00	8.74	5.35	384
Number of additional diagnoses	3.00	3.00	3.24	2.11	289	2.00	0.00	2.07	0.53	94	2.00	2.00	2.96	1.92	384
Months to diagnosis^a^	21.00	44.00	41.33	59.37	228	0.50	0.25	4.87	31.75	91	6.00	35.50	31.77	57.36	320
Number of MDs seen before diagnosis	3.00	2.00	3.73	4.27	227	2.00	0.25	2.22	0.92	90	2.00	1.25	3.30	3.71	317
Number of current MDs treating symptoms	1.00	0.00	1.20	0.73	289	1.00	0.00	1.01	0.1	94	1.00	0.00	1.16	0.64	384
Number of current treatments	3.00	2.00	3.24	1.80	289	1.00	0.00	1.16	0.59	94	2.00	3.00	2.73	1.82	384
Difficulty of receiving a diagnosis	4.00	3.00	3.60	1.36	284	2.00	1.00	1.99	1.03	94	3.00	3.00	3.20	1.46	379
Difficulty of finding a specialist	4.00	2.00	3.74	1.25	282	2.00	1.00	2.06	0.98	94	3.00	3.00	3.33	1.39	377
Perceived understanding of FMS	4.00	1.00	3.65	1.08	274	3.00	0.00	2.98	0.89	87	4.00	1.00	3.49	1.07	362
Perceived understanding of FMS causes	3.00	2.00	3.16	1.11	274	3.00	1.00	2.64	0.72	92	3.00	2.00	3.04	1.06	367
Perceived understanding of FMS from family	3.00	2.00	2.84	1.13	268	4.00	1.00	3.45	1.08	83	3.00	2.00	2.98	1.14	352
Perceived understanding of FMS from MDs	3.00	2.00	2.82	1.27	267	4.00	2.00	3.68	1.13	88	3.00	2.00	3.03	1.29	356
Perceived level of caring from MDs	3.00	2.00	2.75	1.25	275	3.00	1.00	2.66	1.02	88	3.00	2.00	2.74	1.20	364
Perceived level of negative judgment from MDs	3.00	2.00	2.84	1.10	266	3.00	1.00	2.71	0.73	92	3.00	2.00	2.80	1.02	359

*Note.* Md: median; IQR: interquartile range; M: mean; SD: standard deviation; N: number of respondents for item per group.

All variables demonstrated *p* < .001 Shapiro–Wilk Test.

^a^From initial contact with MDs regarding FMS symptoms.

One-sample Wilcoxon signed-rank tests were used to assess whether participants’ clinical experiences significantly deviated from a neutral median on a series of 4- or 5-point scales. Participants reported significantly greater *Difficulty in Receiving a Diagnosis* (Md = 3.00, *n* = 379, *Z* = 2.987, *p* = .003, *r* = 0.15) and *Difficulty in Finding a Specialist* (Md = 3.00, *n* = 377, *Z* = 4.682, *p* < .001, *r* = 0.24) compared to a neutral experience (median of 3). Additionally, participants reported significantly lower *Perceived Level of Caring from MDs* (Md = 3.00, *n* = 364, *Z* = −3.966, *p* < .001, *r* = −0.21) and greater *Perceived Negative Judgement from MDs* (Md = 3.00, *n* = 359, *Z* = 15.415, *p* < .001, *r* = 0.81) compared to a neutral experience (median of 3 and 1, respectively). However, participants reported no significantly different *Perceived Understanding of FMS by MDs* (Md = 3.00, *n* = 356, *Z* = .269, *p* = .788) compared to a neutral experience (median of 3).

### Health information-seeking

**Figure 3.**
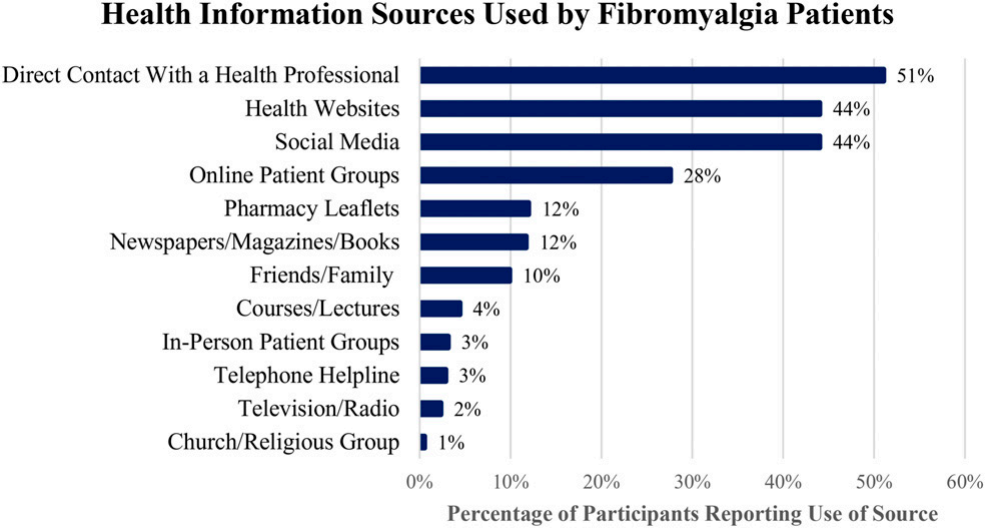
Health information sources used by FMS patients. Bar chart illustrating the distribution of health information sources utilised by FMS patients (*N* = 384). Participants were permitted to select multiple sources. The percentages represent the proportion of respondents reporting accessing each source.

It was found that 84.11% of patients reported actively seeking health information. Direct contact with a health professional (51.0%), health websites (44.0%), social media (44.0%), and online patient groups (27.6%) were the most frequently sought resources for health information. Figure 3 demonstrates the relative frequency of each health information source.

**Figure 4.**
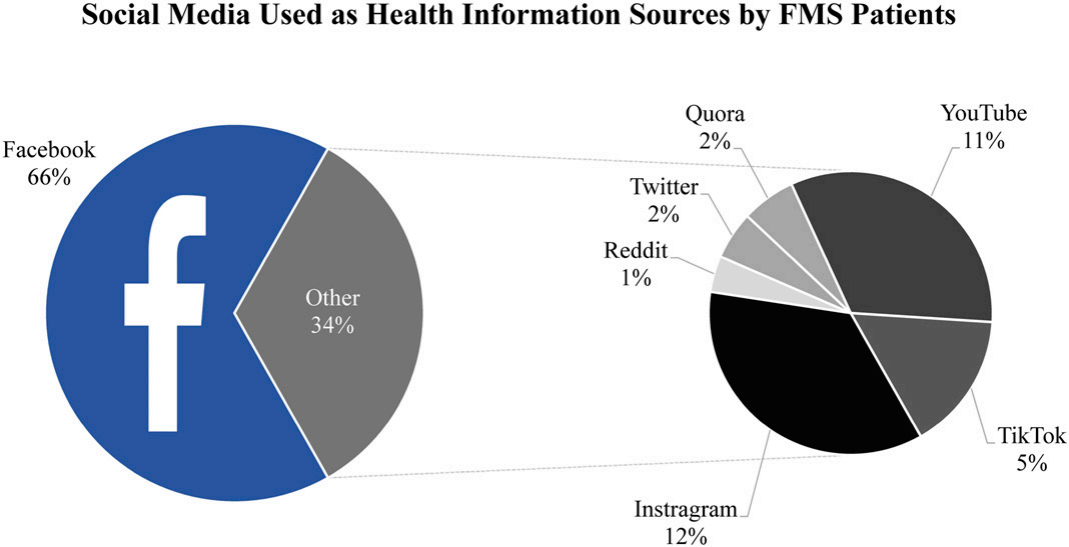
Social media used as health information sources by FMS patients. Pie chart illustrating the proportional usage of social media platforms used by FMS patients as health information sources. Participants were able to select multiple platforms. A total of 435 responses were collected from 384 participants. The exploded pie chart depicts the distribution of lesser used social media platforms.

**Figure 5.**
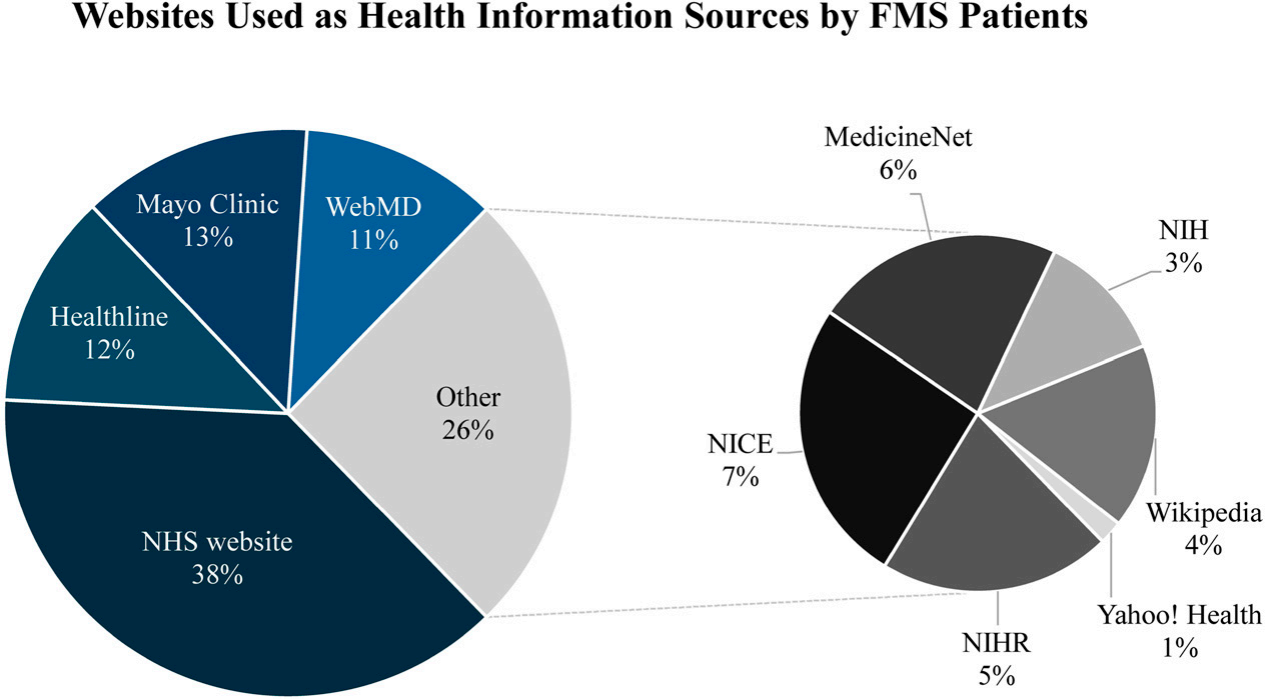
Websites used as health information sources by FMS patients. Pie chart illustrating the distribution of health websites utilised by FMS patients as sources of health information. A total of 731 responses were collected from 384 participants, who were able to select multiple websites. The exploded pie chart highlights the distribution of less frequently accessed websites.

The most frequently used social media platform for health information-seeking was Facebook (67%).^53^ The most frequently used health website was the National Health Service website (38%).^54^ Figures 4 and 5 demonstrate the frequency of each social media site and health website, respectively.

When exploring the reasons for online health information-seeking, 40.6% reported seeking information to decide whether to see a doctor, 28.1% sought information to look up information after an appointment, and 20.8% sought information to prepare for an appointment. Additionally, 232 participants rated their agreement with three statements related to access to health information using a 5-point scale (1 = very much disagree to 5 = very much agree). For the item *‘I often want more health information but I don’t know where to find it’*, 46.6% of participants selected either 4 (somewhat agree) or 5 (very much agree), indicating that nearly half of the sample experienced some difficulty locating health information. The item *‘I expect my doctor/health professionals to provide me with all the information that I need’* was endorsed by 41.4% of participants (ratings of 4 or 5), suggesting a moderate level of expectation that clinicians are responsible for meeting informational needs. Finally, 28.0% of participants agreed or strongly agreed with the statement *‘I often have difficulty finding health information in my primary language’*, indicating that while language-related barriers were less frequently reported, they were still relevant for over a quarter of the sample.

Three hierarchical linear regressions were conducted to further explore the relationship between clinical factors and health information-seeking while accounting for known confounding factors. The first hierarchical model aimed to investigate the relative contribution of clinical factors to predict the *total number of health information sources* used by FMS patients (i.e. pharmacy leaflets, social media, and helpline); the second model focused on the *total number of social media sites* used by FMS patients (i.e. Facebook, X, and Instagram); the third model aimed to predict the *total number of health websites* used by FMS patients (i.e. NHS website, Healthline, and NIHR).

The first model significantly predicted the *total number of health information sources* used by FMS patients *F* (5, 324) = 6.389, *p* < .001, adj. *R*^*2*^ *=* 0.24. After accounting for the effects of age, sex, education, and employment status, three clinical variables contributed significantly to the prediction: *Difficulty in Receiving a Diagnosis* (*p* = .036), *Difficulty in Finding a Specialist* (*p* = .004), and *Perceived Level of Caring from Medical Doctors* (*p =* .007). See Table 2 for regression coefficients, standard errors, and *p-*values.

**Table 2.** Hierarchical regression results for total number of health information sources used.

Number of health information sources used	*B*	95% CI for *B*		*SE B*	β	*p*	*R* ^*2*^	Δ*R*^*2*^	Sig. F change
		*LL*	*UL*						
Model 1							.19	.18	<.001
Constant	3.613	2.59	4.63	.518	-	<.001			
Age	−.001	−0.01	0.01	.007	−.010	.863			
Sex	−1.330	−1.72	−0.93	.199	−.389	<.001			
Education	.234	0.07	0.39	.079	.157	.003			
Employment	−.193	−0.35	−0.02	.084	−.130	.022			
Model 2							.26	.24	<.001
Constant	1.504	−0.03	3.04	.783		.056			
Age	−.007	−0.02	0.00	.007	−.062	.285			
Sex	−.722	−1.15	−0.28	.221	−.211	.001			
Education	.208	0.05	0.35	.077	.14	.007			
Employment	−.177	−0.33	−0.01	.083	−.119	.033			
Diagnostic difficulty	.149	0.01	0.28	.071	.146	.036*			
Difficulty in finding a specialist	.223	0.07	0.37	.077	.211	.004*			
Understanding of MDs	−.035	−0.18	0.11	.076	−.031	.646			
Perceived caring of MDs	.212	0.05	0.36	.078	.173	.007*			
Perceived negative judgement from MDs	−.018	−0.18	0.15	.086	−.013	.831			

*Note*. Model: ‘Enter’ method in SPSS Statistics; *B*: unstandardised regression coefficient; CI: confidence interval; LL: lower limit of confidence interval; UL: upper limit of confidence interval; *SE B*: standard error of the coefficient; β: standardised coefficient; *R*^*2*^: coefficient of determination; Δ*R*^*2*^: adjusted *R*^*2*^.

*p < .05.

Both the second and third models found that clinical factors did not significantly predict the *total number of social media sites* used by FMS patients *F* (5, 324) = 0.540, *p* = .746, adj. *R*^*2*^ *=* 0.006 (see Table 3), nor the *total number of health websites* used by FMS patients *F* (5, 324) 1.919, *p* = .091, adj. *R*^*2*^ *=* 0.130 (see Table 4).

**Table 3.** Hierarchical regression results for total number of social media sites used.

Number of social media sites used	*B*	95% CI for *B*		*SE B*	β	*p*	*R* ^*2*^	Δ*R*^*2*^	Sig. F change
		*LL*	*UL*						
Model 1							.025	.013	.084
Constant	2.108	1.46	2.75	.328		<.001			
Age	−.01	−0.01	−0.00	.004	−.146	.022			
Sex	−.193	−0.44	0.05	.126	−.098	.126			
Education	−.016	−0.11	0.08	.05	−.019	.745			
Employment	−.08	−0.18	0.02	.053	−.094	.132			
Model 2							.033	.006	.746
Constant	1.919	0.90	2.93	.518		<.001			
Age	−.01	−0.01	−0.00	.004	−.148	.026			
Sex	−.122	−0.41	0.16	.146	−.062	.405			
Education	−.02	−0.12	0.08	.051	−.023	.695			
Employment	−.067	−0.17	0.04	.055	−.079	.219			
Diagnostic difficulty	−.008	−0.10	0.08	.047	−.013	.872			
Difficulty in finding a specialist	.047	−0.05	0.14	.051	.077	.36			
Understanding of MDs	−.014	−0.11	0.08	.05	−.021	.782			
Perceived caring of MDs	−.016	−0.11	0.08	.052	−.023	.753			
Perceived negative judgement from MDs	.016	−0.09	0.12	.057	.019	.786			

*Note*. Model: ‘Enter’ method in SPSS Statistics; *B*: unstandardised regression coefficient; CI: confidence interval; LL: lower limit of confidence interval; UL: upper limit of confidence interval; *SE B*: standard error of the coefficient; β: standardised coefficient; *R*^*2*^: coefficient of determination; Δ*R*^*2*^: adjusted *R*^*2*^.

**Table 4.** Hierarchical regression results for total number of health sites used.

Number of health sites used	*B*	95% CI for *B*		*SE B*	β	*p*	*R* ^*2*^	Δ*R*^*2*^	Sig. F change
		*LL*	*UL*						
Model 1							.129	.118	<.001
Constant	3.098	1.90	4.29	.606	-	<.001			
Age	.002	−0.01	0.01	.008	.016	.786			
Sex	−1.266	−1.72	−0.80	.233	−.328	<.001			
Education	.228	0.04	0.41	.093	.135	.014			
Employment	−.112	−0.30	0.08	.098	−.067	.254			
Model 2							.154	.130	.091
Constant	2.199	0.33	4.06	.946	-	.021			
Age	−.002	−0.01	0.01	.008	−.012	.847			
Sex	−.873	−1.39	−0.34	.267	−.226	.001			
Education	.214	0.03	0.39	.093	.127	.022			
Employment	−.084	−0.28	0.11	.10	−.05	.401			
Diagnostic difficulty	.08	−0.08	0.24	.086	.07	.349			
Difficulty in finding a specialist	.143	−0.04	0.32	.093	.119	.127			
Understanding of MDs	−.085	−0.26	0.09	.092	−.067	.353			
Perceived caring of MDs	.067	−0.11	0.25	.094	.049	.476			
Perceived negative judgement from MDs	−.041	−0.24	0.16	.104	−.025	.697			

*Note*. Model: ‘Enter’ method in SPSS Statistics; *B*: unstandardised regression coefficient; CI: confidence interval; LL: lower limit of confidence interval; UL: upper limit of confidence interval; *SE B*: standard error of the coefficient; β: standardised coefficient; *R*^*2*^: coefficient of determination; Δ*R*^*2*^: adjusted *R*^*2*^.

## Discussion

The main objective of this study was to examine patterns of health information-seeking among FMS patients and to investigate how clinical experiences are associated with these behaviours. As hypothesised, the findings demonstrated high levels of active health information-seeking and show that patients draw on both non-digital and digital resources. Importantly, clinical experiences, both positive and negative, were associated with the breadth of information sources sought, providing partial support for the study hypothesis.

Consistent with previous research in chronic pain populations,^55–57^ participants reported significant dissatisfaction in receiving diagnoses and accessing specialist care. Although the median time to diagnosis in this sample was shorter than reported in earlier UK studies,^10,11,36,58^ patients nonetheless perceived the diagnostic process as difficult and reported barriers to treatment. This suggests that FMS patients continue to experience unmet clinical needs. Challenges associated with the clinical pathway appear to meaningfully shape engagement with health information, as greater difficulty receiving a diagnosis and finding a specialist were associated with consulting a wider range of information sources.

Although participants reported substantial perceived negative judgement from healthcare professionals, including a large effect size (*r* = 0.81), this factor did not independently predict health information-seeking behaviour. This suggests that while negative judgement is a salient feature of the clinical experience for many people with FMS, its influence on information-seeking behaviour may be indirect or mediated by other aspects of care.

Notably, perceived level of caring from medical doctors was also a significant predictor of the number of information sources used. This finding indicates that health information-seeking may also reflect proactive engagement encouraged by supportive clinical relationships. Positive patient–provider interactions have been shown to foster trust, empowerment,^59–61^ and collaborative approaches to health management.^62–64^ By quantifying these interactions and associating them with health information-seeking, this study advances our understanding of the relationship between clinical relationships and patient behaviours; feeling cared for may motivate patients to seek information more actively and to integrate multiple sources alongside professional advice, rather than disengaging from care. Merkley^65^ found that patients with long-term health issues tended to trust their healthcare providers and felt empowered when seeking health information online, but they expressed frustration with trying to access high-quality and trustworthy information. Healthcare professionals can offer support by endorsing trustworthy resources, guiding patients toward evidence-based information, and fostering collaborative health discussions.

Findings related to specific health information sources provide novel and practically relevant insights, with 84% actively seeking health information. Direct contact with healthcare professionals remained the most frequently used source of information (51%), underscoring the central role of clinicians in patients’ information networks. However, digital sources were also widely used, with 44% of participants accessing both health website and social media. The NHS website was the most commonly used health website (38%), suggesting trust in authoritative and institutionally endorsed sources, while Facebook was the dominant social media platform (67%), reflecting its role in facilitating peer support and condition-specific communities.^66^ These findings highlight the growing importance of digital platforms alongside traditional care. Emerging research points to the growing importance of digital health technologies in chronic pain management, including both peer-support interventions and clinician-supported tools.^67,68^ While digital engagement offers clear benefits, it also exposes patients to risks such as misinformation, unlicensed alternate treatments, and fraudulent health products.^69–72^ This highlights the need for improved regulation and oversight to ensure the quality of online health information.^72,73^ Accordingly, health communication strategies should evolve to promote the benefits of information-seeking as well as supporting patients in evaluating the credibility of sources. Strengthening digital health literacy alongside the provision of trustworthy resources would maximise the benefits of increasingly hybrid information environments.

Despite high levels of health information-seeking in this sample, participants reported persistent barriers to accessing the information they need. Nearly half (108/232) indicated difficulty knowing where to find health information, and over 40% expected healthcare professionals to provide necessary information. This highlights a potential mismatch between patient expectations and the realities of time-limited clinical encounters. Additionally, over a quarter of participants reported difficulty accessing information in their primary language, pointing to accessibility issues may exacerbate health inequalities.

Taken together, these findings suggest that health information-seeking among people with FMS is a multifaceted behaviour shaped by clinical experiences, relational factors, and the accessibility of both digital and non-digital resources. Digital platforms appear to function as complementary rather than alternative sources of information, particularly when traditional care pathways are perceived as insufficient or when supportive clinician relationships encourage shared information-seeking.

Future research should prioritise longitudinal designs to empirically examine the causal links between patient–provider interactions and health outcomes over time. Additionally, qualitative inquiry is required to explore how patients construct their understanding of FMS, specifically regarding the cognitive and emotional dimensions of symptom management. Studies should also compare health information-seeking behaviours between patients with high versus low digital engagement. Finally, research should assess how evolving clinical frameworks, like the classification of FMS as a primary pain syndrome^74^ and recently updated guidance on multidisciplinary programmes,^75^ impact patient–provider dynamics, specifically regarding communication, trust, and shared decision-making.

In practice, interventions must go beyond mitigating negative experiences to actively fostering empowering interactions. Provider training should prioritise compassionate communication and shared decision-making to enhance patient trust. From a policy perspective, ensuring access to clear, evidence-based guidance is essential to reduce reliance on unverified sources and enhance patient autonomy. Crucially, information dissemination strategies must be inclusive, ensuring equitable access to trustworthy resources regardless of a patient’s digital literacy.

### Limitations

Although the sample size was adequate for the planned analyses, the findings may not be fully generalisable to the wider UK FMS population. Recruitment was conducted primarily through social media platforms and via patients known to the research team, which may have resulted in a sample that was more digitally engaged than the broader FMS population. Individuals who rely predominantly on offline sources of information may therefore be underrepresented. Key demographic variables such as socioeconomic status were not collected, limiting assessment of representativeness. Additionally, the study did not assess health anxiety or related mental health factors, which may influence patterns of health information-seeking; the absence of such measures limits the ability to determine whether observed associations reflect clinical experiences, underlying psychological factors, or an interaction between the two.

The study relied on self-reported diagnosis of FMS. Although participants were required to confirm a formal diagnosis and indicate the diagnosing clinician, medical records were not verified. While common in online survey research, this approach may introduce misclassification bias. Furthermore, survey items were not formally piloted. Although questions were informed by previous literature and chronic pain research, lack of piloting may have affected clarity or contributed to response fatigue.

Language-related findings should be interpreted cautiously, as primary language was not directly assessed in the demographic data. Consequently, the proportion of participants whose primary language was not English is unknown, limiting interpretation of reported difficulty accessing information in one’s primary language.

The absence of a qualitative component limits the ability to fully understand the motivations and contextual factors driving these health information-seeking behaviours. Future research would benefit from integrating qualitative methodologies and patient and public involvement (PPI) at the survey development stage to ensure greater relevance, depth, and sensitivity to the lived experiences of FMS patients. Finally, given the cross-sectional nature of the study, causal inferences cannot be drawn. The findings highlight relevant associations that warrant further investigation through research specifically designed to examine causal pathways and underlying mechanisms.

### Conclusion

This study provides quantitative evidence that specific diagnostic and treatment experiences are associated with the extent of health information-seeking among people with FMS. Greater difficulty receiving a diagnosis and accessing specialist care were linked to engagement with a wider range of health information sources. Notably, higher perceived caring from medical doctors was also associated with greater information-seeking, suggesting that supportive clinical relationships may encourage proactive engagement with health information.

By examining these associations across both digital and non-digital sources, this study extends previous qualitative findings and highlights the importance of clinical experiences in shaping how patients navigate health information. Strengthening patient–provider communication and effective signposting to credible resources may support informed and collaborative care.

## Supplemental material

Supplemental material - Impact of fibromyalgia syndrome diagnosis and treatment experiences on health information-seeking behaviour: A cross-sectional online survey Supplemental material for Impact of fibromyalgia syndrome diagnosis and treatment experiences on health information-seeking behaviour: A cross-sectional online survey by Juliette Allen, Andreas Goebel, and Nicholas Fallon in British Journal of Pain

Supplemental material - Impact of fibromyalgia syndrome diagnosis and treatment experiences on health information-seeking behaviour: A cross-sectional online survey Supplemental material for Impact of fibromyalgia syndrome diagnosis and treatment experiences on health information-seeking behaviour: A cross-sectional online survey by Juliette Allen, Andreas Goebel, and Nicholas Fallon in British Journal of Pain

## Data Availability

Data is available from the authors on request.*
