# Supplementary material for: Impact of fibromyalgia syndrome diagnosis and treatment experiences on health information-seeking behaviour: A cross-sectional online survey
Source: Br J Pain. 2026 May 6:20494637261447443. Online ahead of print. doi: 10.1177/20494637261447443 (PMC13149355; doi:10.1177/20494637261447443)
Supplement: Supplemental material - Impact of fibromyalgia syndrome diagnosis and treatment experiences on health information-seeking behaviour: A cross-sectional online survey [file sj-pdf-1-bjp-10.1177_20494637261447443.pdf]

Supplementary Materials – Copy of Survey

Section 1: Demographics

- **How old are you?**
  [please enter in years]
- **What is your sex?**
  [select one: Female, Male, Prefer not to say, Other {please enter}]
- **What is your highest level of education?**
  [select one: No qualifications, GCSE, A-Level/B-Tech, Bachelor's degree, Post-graduate level]
- **What is your employment level?**
  [select one: unemployed, part-time employed, full-time employed, self-employed]
- **What is your ethnic group?**
  [select one: Asian/Asian British, Black/African/Caribbean/Black British, Mixed/Multiple Ethnic Groups, White, Other {please enter}]

Section 2: FMS Symptoms & Experience

- **Please select which of the following symptoms of Fibromyalgia you have experienced.**
  [select multiple: chronic widespread pain, problems sleeping, fatigue, headaches, heightened sensitivity to touch, sensitivity to pressure, difficulty concentrating, numbness and/or tingling, feelings of anxiety, feelings of depression, joint pain, stiffness, low back pain, other {please enter}]
- **How long ago did you first notice the widespread symptoms of Fibromyalgia?**
  [please enter years/months]
- **In the past week, how severe have the following symptoms been on average?**
  - **Chronic Widespread Pain**
    [Likert scale 0-10]
  - **Fatigue**
    [Likert scale 0-10]
- **Have you experienced any regional chronic pains before you developed widespread pain and Fibromyalgia symptoms?**
  [select one: yes {please enter}, no]
- **Fibromyalgia flares/flare ups are when symptoms increase in intensity for a period, usually lasting days or weeks. Flares are noticeably different to your everyday experience of fibromyalgia.**
  How often do you experience FMS flares?
  [please enter every … X … weeks, or select N/A]
- **How often do your FMS flares last?**
  [please enter days, or select N/A]
- **What treatments are you currently using to treat your FMS?**
  [select multiple: pain relievers over the counter without prescription, pain relievers prescribed by your medical doctor, other drugs prescribed by your medical doctor, sleeping aids, any talking-based therapy, relaxation techniques, biofeedback, lifestyle changes, exercise, none, other {please enter}]

Section 3: FMS Diagnosis

- **How long did it take you to see a medical doctor after you first experienced any widespread pain from Fibromyalgia?**
  [please enter year/months/weeks]
- **From the time you first saw a physician about the symptoms you were experiencing, how long did it take before you were diagnosed with Fibromyalgia?**
  [please enter year/months/weeks]
- **How many physicians did you see about your widespread pain until you received your diagnosis of Fibromyalgia?**
  [please enter number]
- **From the time you first saw a medical doctor about your widespread symptoms and up until now, please select what types of doctors you saw about your symptoms of Fibromyalgia.**
  [select multiple: General Practitioner, Rheumatologist, Pain Specialist, Physiotherapist, Gynaecologist, Internist, Neurologist, Psychiatrist, Orthopaedic Surgeon, Anaesthesiologist, Not Sure, Other {please enter}]
- **What type of medical doctor diagnosed you with Fibromyalgia?**
  [select one: General Practitioner, Rheumatologist, Pain Specialist, Physiotherapist, Gynaecologist, Internist, Neurologist, Psychiatrist, Orthopaedic Surgeon, Anaesthesiologist, Not Sure, Other {please enter}]
- **What kind of medical doctor is currently treating your Fibromyalgia?**
  [select multiple: General Practitioner, Rheumatologist, Pain Specialist, Physiotherapist, Gynaecologist, Internist, Neurologist, Psychiatrist, Orthopaedic Surgeon, Anaesthesiologist, Not Sure, Other {please enter}]
- **Overall, how easy/difficult was your experience in receiving a Fibromyalgia diagnosis?**
  [select one: Very easy, somewhat easy, neither easy nor difficult, somewhat difficult, very difficult]
- **Which of the following localised pain conditions, if any, were you diagnosed with before being told you have Fibromyalgia?**
  [select multiple: Mechanical Low Back Pain, Sciatica, Pelvic Pain, None, Other {please enter}]
- **Which of the following, if any, were you diagnosed with before being told you have Fibromyalgia?**
  [select multiple: Osteoarthritis, Irritable Bowl Syndrome, Chronic Fatigue Syndrome, Rheumatoid Arthritis, Systemic Lupus Erythematosus, Ankylosing Rheumatic, Polymyalgia Rheumatic, Myositis Myopathies, Hypothyrdoisim, Neuropathies, Temporomandibular Disorders, Multiple Chemical Sensitivities, Tension and Migraine Headache, Interstitial Cystitis, Other {please enter}]
- **Overall, how easy/difficult was your experience in identifying a specialist to treat your Fibromyalgia?**
  [select one: Very easy, somewhat easy, neither easy not difficult, somewhat difficult, very difficult]

Section 4: Understanding

- **How well do you feel you understand Fibromyalgia Syndrome?**
  [select one: Very well, somewhat well, neither well nor poorly, somewhat poorly, very poorly]
- **How well do you understand the *underlying* causes of Fibromyalgia?**
  [select one: Very well, somewhat well, neither well nor poorly, somewhat poorly, very poorly]
- **How have your family and friends understood your Fibromyalgia?**
  [select one: Very well, somewhat well, neither well nor poorly, somewhat poorly, very poorly]
- **How well have your medical doctors understood your Fibromyalgia?**
  [select one: Very well, somewhat well, neither well nor poorly, somewhat poorly, very poorly]
- **How caring do you feel the medical doctors you have seen about your Fibromyalgia symptoms have been?**
  [select one: Very caring, somewhat caring, neither caring nor uncaring, somewhat uncaring, very uncaring]
- **In the last 12 months, how often have you felt that a doctor negatively judged you because of your diagnosis?**
  [select one: Always, Often, Sometimes, Never]

<u>Section 5: Health Information</u>

- **Which of the following sources do you use to get information about Fibromyalgia Syndrome?**
  [select multiple: Direct contact with a health professional, pharmacy leaflets at the doctors' office, telephone helpline, television/radio, newspapers/magazines/books, friends/family, church/religious group, courses/lectures, patient groups (in person), patient groups (online), social media, health websites, other {please enter}]
- **Which social media sites do you use to get your health information from?**
  [select multiple: Facebook, Instagram, Reddit, Twitter, Quora, Youtube, Tiktok, not applicable, other {please enter}]
- **Which health websites do you use to get your health information from?**
  [select multiple: NHS website, Healthline, National Institute of Health Research, National Institute for Health and Care Excellence, Mayo Clinic, MedecineNet, National Institutes of Health, WebMD, Wikipedia, Yahoo! Health, not applicable, other {please enter}]
- **Are you aware of some very recent scientific research from Kings College London and the University of Liverpool which demonstrates that Fibromyalgia Syndrome could be an Autoimmune Disease?**
  [select one: Yes or No]
