# Supplementary material for: Impact of fibromyalgia syndrome diagnosis and treatment experiences on health information-seeking behaviour: A cross-sectional online survey
Source: Br J Pain. 2026 May 6:20494637261447443. Online ahead of print. doi: 10.1177/20494637261447443 (PMC13149355; doi:10.1177/20494637261447443)
Supplement: Supplemental material - Impact of fibromyalgia syndrome diagnosis and treatment experiences on health information-seeking behaviour: A cross-sectional online survey [file sj-pdf-2-bjp-10.1177_20494637261447443.pdf]

GROUP DIFFERENCES

APPROACH TO DATA ANALYSIS

Given violations of normality assumptions for most continuous variables, all group comparisons were conducted using non-parametric statistical methods.

To examine sex differences across the key study variables, a series of non-parametric Wilcoxon rank-sum tests were conducted. The one non-binary participant was excluded because the Wilcoxon rank-sum test assumes two independent groups. For each variable, the Wilcoxon test statistic (*W*), standardised *z*-score, and corresponding *p*-value were reported. Effect sizes (*r*) were calculated using the formula $r = Z / \sqrt{N}$, where *N* is the total number of observations included in the comparison. Group medians and interquartile ranges are reported in Table 1 in the main paper; statistical results are presented below in Table S1.

Group differences across education attainment groups (No Qualifications, *n* = 16; GCSE, *n* = 79; A-Level, *n* = 141; Undergraduate Degree, *n* = 115; Postgraduate Degree, *n* = 5) and across employment groups (Unemployed, *n* = 145; Part-time Employed, *n* = 62; Full-time Employed, *n* = 153; Self-Employed, *n* = 24) were evaluated using Kruskal-Wallis H tests. For outcome variables showing significant omnibus results, Dunn's pairwise post-hoc tests with Bonferroni correction were conducted. Effect sizes for pairwise comparisons were calculated using the same method as above ($r = Z / \sqrt{(n1 + n2)}$, where *n1* and *n2* are group sizes). Results from the Kruskal-Wallis tests for education are shown in Table S2, and for employment in Table S4. Descriptive statistics including medians and interquartile ranges are reported in Table S3 for education and Table S5 for employment.

RESULTS

Differences Between the Sexes

Female participants reported significantly greater *Duration of FMS Flares (days)* (*W*= 39,331, *Z*= 7.63, *p*<.001, *r*= 0.43), *CWP Severity* (*W*= 60,442, *Z*= 6.53, *p*<.001, *r*= 0.34), *Fatigue Severity* (*W*= 61,321.5, *Z*= 8.14, *p*<.001, *r*= 0.42), *Number of Symptoms* (*W*= 67,166, *Z*= 12.74, *p*<.001, *r*= 0.65), *Number of Additional Diagnoses* (*W*= 60,069.5, *Z*= 5.15, *p*<.001, *r*= 0.26), *Months to Diagnosis* (*W*= 44,174, *Z*= 10.38, *p*<.001, *r*= 0.58), *Number of MDs Seen Before Diagnosis* (*W*= 38,960, *Z*= 4.05, *p*<.001, *r*= 0.23), *Number of Current Treatments* (*W*= 65,446.5, *Z*= 10.98, *p*<.001, *r*= 0.56), *Difficulty Receiving a Diagnosis* (*W*= 62,039.5, *Z*= 9.17, *p*<.001, *r*= 0.47), *Difficulty Finding a Specialist* (*W*= 62,087.5, *Z*= 10.03, *p*<.001, *r*= 0.52), and *Perceived Understanding of FMS* (*W*= 54,246, *Z*= 5.72, *p*<.001, *r*= 0.30) compared to male participants.

Male participants reported significantly greater *Frequency of FMS Flares (weeks)* (*W*= 40,581, *Z*= 3.85, *p*<.001, *r*= 0.21), *Perceived Understanding from Family* (*W*= 43,879.5, *Z*= 4.21, *p*<.001, *r*= 0.22), and *Perceived Understanding from MDs* (*W*= 43,128, *Z*= –5.39, *p*<.001, *r*= 0.29) compared to female participants.

Despite reporting the same median and interquartile ranges, there was a significant difference between female and male participants in *Number of Current MDs Treating FMS* (*W*= 56,958.5, *Z*= 2.33, *p*=.020, *r*= 0.12), *Perceived Understanding of FMS Causes* (*W*= 54,031.5, *Z*= 4.44, *p*<.001, *r*= 0.23), *Total Sources of Information Sought* (*W*= 62,798.5, *Z*= 8.16, *p*<.001, *r*= 0.42), and *Total Health Websites Sought* (*W*= 60,696, *Z*= 5.95, *p*<.001, *r*= 0.30). Boxplots indicated a larger and higher distribution among female participants compared to male participants for all variables.

Differences Between Educational Attainment Groups

There was a significant difference in *CWP Severity* ($\chi^2(4)= 36.77$, $p<.001$) and *Fatigue Severity* ($\chi^2(4)= 36.77$, $p<.001$) between educational attainment groups; participants with No Qualification reported significantly greater *CWP Severity* compared to participants with A-Level Attainment ($Z= 3.69$, $p=.002$, $r= 0.29$), UG Degree Attainment ($Z= 4.88$, $p<.001$, $r= 0.43$), and PG Degree Attainment ($Z= 3.96$, $p=.001$, $r= 0.57$); participants with GSCE Attainment reported significantly greater *CWP Severity* compared to participants with UG Degree Attainment ($Z= 4.36$, $p<.001$, $r= 0.32$). Participants with No Qualifications also reported significantly greater *Fatigue Severity* compared to participants with A-Level Attainment ($Z= 3.16$, $p=.016$, $r= 0.25$), UG Degree Attainment ($Z= 4.73$, $p<.001$, $r= 0.42$), and PG Degree Attainment ($Z= 3.29$, $p=.010$, $r= 0.48$); participants with GCSE Attainment ($Z= 4.07$, $p<.001$, $r= 0.30$) and A-Level Attainment ($Z= 3.58$, $p=.003$, $r= 0.23$) reported significantly greater *Fatigue Severity* compared to UG Degree Attainment.

There was a significant difference in *Number of Symptoms* ($\chi^2(4)= 37.46$, $p<.001$) between educational attainment groups; participants with No Qualifications reported significantly greater *Number of Symptoms* compared to participants with A-Level Attainment ($Z= 3.12$, $p=.018$, $r= 0.25$) and UG Degree Attainment ($Z= 4.05$, $p=.001$, $r= 0.35$); participants with GCSE Attainment also reported significantly greater *Number of Symptoms* compared to with A-Level Attainment ($Z= 3.53$, $p=.004$, $r= 0.24$) and UG Degree Attainment ($Z= 5.15$, $p<.001$, $r= 0.37$).

There was a significant difference in *Months to Diagnosis* ($\chi^2(4)= 17.03$, $p= .002$) and *Number of Current Treatments* ($\chi^2(4)= 13.66$, $p= .008$) between educational attainment groups; participants with GCSE Attainment reported significantly greater *Months to Diagnosis* ($Z= 3.73$, $p=.002$, $r= 0.24$) and *Number of Current Treatments* ($Z= 2.99$, $p=.028$, $r= 0.21$) compared to participants with UG Degree Attainment.

There was a significant difference in *Difficulty in Finding a Specialist* ($\chi^2(4)= 18.88$, $p<.001$) between educational attainment groups; participants with No Qualifications reported significantly greater difficulty compared to participants with A-Level Attainment ($Z= 3.50$, $p=.005$, $r= 0.28$) and PG Degree Attainment ($Z= 3.51$, $p=.005$, $r= 0.31$).

There was a significant difference in *Perceived Understanding of FMS Causes* ($\chi^2(4)= 13.44$, $p= .009$) between educational attainment groups; participants with GCSE Attainment reported significantly greater difficulty compared to participants with A-Level Attainment ($Z= 3.03$, $p=.024$, $r= 0.21$) and PG Degree Attainment ($Z= 2.97$, $p=.029$, $r= 0.22$).

Between educational attainment groups, there were also significant differences in *Duration of Flares (days)* ($\chi^2(4)= 13.91$, $p=.008$), *Number of Additional Diagnoses* ($\chi^2(4)= 9.81$, $p= .044$), and *Difficulty in Receiving a Diagnosis* ($\chi^2(4)= 13.68$, $p= .008$). However, post-hoc tests revealed no significant pairwise comparisons (ps=.07-.55).

Differences Between Employment Groups

There was a significant difference in *Frequency of FMS flares (weeks)* ($\chi^2(3)= 14.8$, $p=.002$) and *Duration of FMS flares (days)* ($\chi^2(3)= 44.68$, $p<.001$) between employment groups; Unemployed participants reported lower *Frequency* ($Z= 3.58$, $p=.002$, $r= 0.22$) but greater *Duration* ($Z= 6.37$, $p<.001$, $r= 0.41$) *of Flares* compared to participants that were FT Employed. PT Employed ($Z= 2.72$, $p=.039$, $r= 0.20$) and Self-Employed ($Z= 3.31$, $p=.006$, $r= 0.26$) participants reported greater *Duration of Flares* compared to FT Employed participants.

There was a significant difference in *CWP Severity* $\chi^2(3)= 73.01$, $p<.001$) and *Fatigue Severity* ($\chi^2(3)= 69.17$, $p<.001$) between employment groups; Unemployed participants reported significantly greater

CWP (*Z*= 4.28, *p*<.001, *r*= 0.30) and *Fatigue* (*Z*= 8.47, *p*<.001, *r*= 0.49) *Severity* compared to FT Employed participants, as well as greater *CWP Severity* compared to PT Employed participants (*Z*= 8.29, *p*<.001, *r*= 0.48). PT Employed participants also reported significantly greater *Fatigue Severity* compared to FT Employed participants (*Z*= 3.75, *p*=.001, *r*= 0.26).

There was a significant difference in *Number of Symptoms* ($\chi^2(3)$= 133.24, *p*<.001) between employment groups; Unemployed participants reported significantly greater *Number of Symptoms* compared to PT Employed participants (*Z*= 3.22, *p*=.008, *r*= 0.22). Additionally, Unemployed (*Z*= 11.03, *p*<.001, *r*= 0.64), PT Employed (*Z*= 5.25, *p*<.001, *r*= 0.36), and Self-Employed (*Z*= 6.03, *p*<.001, *r*= 0.45) participants all reported significantly greater *Number of Symptoms* compared to FT Employed participants.

There was a significant difference in *Number of Additional Diagnoses* ($\chi^2(3)$= 12.48, *p*=.006) between employment groups; Unemployed participants reported significantly greater *Number of Additional Diagnoses* compared to FT Employed participants (*Z*= 3.52, *p*=.003, *r*= 0.20).

There was a significant difference in *Months to Diagnosis* ($\chi^2(3)$= 72.42, *p*<001) between employment groups; compared to FT Employed participants, Unemployed (*Z*= 8.61, *p*<.001, *r*= 0.55), PT Employed (*Z*= 4.19, *p*<.001, *r*= 0.30), and Self-Employed (*Z*= 3.88, *p*=.008, *r*= 0.30) participants reported significantly greater *Months to Diagnosis.*

There was a significant difference in *Number of Current MDs Treating FMS* ($\chi^2(3)$= 8.43, *p*=.038) between employment groups; Unemployed participants reported significantly more compared to FT Employed participants (*Z*= 2.83, *p*=.028, *r*= 0.16).

There was a significant difference in *Number of Current Treatments* ($\chi^2(3)$= 65.91, *p*<.001) between employment groups; Unemployed (*Z*= 7.26, *p*<.001, *r*= 0.42), PT Employed (*Z*= 5.09, *p*<.001, *r*= 0.35), and Self-Employed (*Z*= 4.62, *p*<.001, *r*= 0.35) participants reported significantly greater *Number of Current Treatments* compared to FT Employed participants.

There was a significant difference in *Difficulty Receiving a Diagnosis* ($\chi^2(3)$= 45.73, *p*<.001) and *Difficulty Finding a Specialist* ($\chi^2(3)$= 52.51, *p*<.001) between employment groups; Unemployed (*Z*=6.64, *p*<.001, *r*= 0.39) and PT Employed (*Z*= 3.45, *p*=.008, *r*= 0.24) participants reported significantly greater *Difficulty Receiving a Diagnosis* compared to FT Employed participants, as well as *Difficulty Finding a Specialist* (*Z*= 6.84, *p*<.001, *r*= 0.40; *Z*= 3.98, *p*<.001, *r*= 0.27, respectively). Additionally, Self-Employed participants (*Z*= 3.66, *p*=.008, *r*= 0.28) reported significantly greater *Difficulty Finding a Specialist* compared to FT Employed participants.

There was a significant difference in *Perceived Understanding of FMS* ($\chi^2(3)$= 28.89, *p*<.001) and *Perceived Understanding of FMS Causes* ($\chi^2(3)$= 26.50, *p*<.001) between employment groups; for both variables, Unemployed (*Z*= 4.74, *p*<.001, *r*= 0.28; *Z*= 4.79, *p*<.001, *r*= 0.29, respectively), PT Employed (*Z*= 3.55, *p*=.002, *r*= 0.25; *Z*= 2.95, *p*=.019, *r*= 0.20, respectively), and Self-Employed (*Z*= 3.03, *p*=.015, *r*= 0.23; *Z*= 2.67, *p*=.046, *r*= 0.20, respectively) participants reported greater scores compared to FT Employed participants.

There was a significant difference in *Perceived Understanding of FMS from MDs* ($\chi^2(3)$= 26.53, *p*<.001) between employment groups; FT Employed participants reported significantly greater scores compared to Unemployed participants (*Z*= 5.11, *p*<.001, *r*= 0.31).

There was a significant difference in *Perceived Negative Judgement from MDs* ($\chi^2(3)= 16.30$, $p<.001$) between employment groups; Unemployed participants reported significantly greater scores compared to PT Employed ($Z= 3.69$, $p=.001$, $r= 0.27$) and FT Employed ($Z= 2.76$, $p=.035$, $r= 0.17$) participants.

There was a significant difference in *Number of Total Sources Sought* ($\chi^2(3)= 41.10$, $p<.001$) and *Number of Health Websites Sought* ($\chi^2(3)= 21.11$, $p<.001$) between employment groups; for both variables, Unemployed ($Z= 5.34$, $p<.001$, $r= 0.31$; $Z= 3.06$, $p=.013$, $r= 0.18$, respectively), PT Employed ($Z= 3.96$, $p<.001$, $r= 0.27$; $Z= 3.18$, $p=.009$, $r= 0.22$, respectively), and Self-Employed ($Z= 4.34$, $p<.001$, $r= 0.33$; $Z= 3.56$, $p=.002$, $r= 0.27$, respectively) participants reported greater scores compared to FT Employed participants.

Between employment groups, there were also significant differences in *Perceived Understanding of FMS from Family* ($\chi^2(3)= 9.89$, $p=.019$). However, post-hoc tests revealed no significant pairwise comparisons (*ps*=.08-.22).

Exploratory Scientific Awareness Findings

This study also investigated the quality of health information patients were exposed to and the impact of scientific discoveries in this community. Participants were asked if they had heard about a recent novel study that gained widespread media attention in the year preceding our survey. The study in question was recent collaborative work from the University of Liverpool, Kings College London, and the Karolinska Institute, which offers evidence that FMS could be an autoimmune condition[1,2]. This study was chosen because of its wide distribution across academic and non-academic media, including pieces in The Guardian[3], The Pain Relief Foundation[4], The Scientist[5], and Healthline[6].

It was found that of 131 patients who responded, 60.31% reported having heard of the research, whilst 39.69% had not. Of 57 Patients who reported where they had heard of this research, 35.09% had heard of the study from social media and 17.54% had heard of the study through online patient groups. Of 22 patients who reported which social media channels they had heard of the study, 77.27% reported hearing of the study on Facebook.

These findings indicate a notable level of engagement with high-quality scientific research. Patients actively seeking high-quality research demonstrate a desire for knowledge, empowerment, and involvement in their healthcare[7–9].

DISCUSSION

This study found a range of significant group differences across sex, educational attainment, and employment status in participants' clinical and diagnostic experiences of FMS. These findings highlight potential disparities in symptom burden, healthcare experiences, and diagnostic. However, given the cross-sectional design and self-reported nature of the data, these results should be interpreted cautiously.

Participants' experiences differed notably by sex, with female participants reporting significantly greater symptom burden and diagnostic challenges compared to male participants. Specifically, women reported a higher number of symptoms, longer time to diagnosis, a greater number of treatments, and more difficulty in finding a specialist, all with large effect sizes ($r>.5$). These patterns are consistent with broader literature on gender disparities in chronic pain conditions, which suggests that women often face more complex clinical presentations[1,2], are more likely to have their symptoms under-recognised or dismissed[3,4], and may encounter gendered biases within healthcare settings[5–7].

Educational attainment emerged as another correlate of clinical severity. Participants with no formal qualifications reported higher chronic widespread pain scores compared to those with postgraduate degrees, alongside a greater number of overall symptoms. Those with higher education also tended to report shorter diagnostic delays and fewer barriers to accessing specialist care, although not all effects reached significance after correction. These findings align with existing evidence linking lower educational attainment to poorer health outcomes across a range of conditions[8–10], often explained through pathways such as reduced health literacy, limited access to resources, and broader socioeconomic disadvantage.

Employment status was similarly associated with differences in symptom burden and diagnostic experiences. Full-time employed participants reported fewer months to diagnosis than those in other employment categories. Moreover, unemployed participants reported the highest number of symptoms, more severe fatigue, and greater chronic widespread pain compared to those employed full-time, with some medium-to-large effect sizes. One interpretation of these findings is that employment may confer structural advantages that facilitate healthcare access, such as financial resources and social support networks. These factors may enable earlier and more effective engagement with healthcare providers, leading to faster diagnosis and potentially more manageable symptom progression. Conversely, individuals with severe or prolonged symptoms may struggle to maintain employment, particularly in full-time roles, resulting in a higher burden of symptoms within unemployed groups.

Collectively, these findings underscore the complex interplay between social factors and the clinical and diagnostic experiences of FMS patients. While certain groups appear to face more substantial burdens and barriers, the directionality of these relationships remains unclear. It is plausible that social inequalities shape healthcare access and symptom management, but equally that the experience of living with severe and poorly managed FMS symptoms constrains educational and employment opportunities. To better understand these dynamics, future research should employ longitudinal designs that can capture the temporal relationships between sociodemographic factors, symptom trajectories, healthcare access, and functional outcomes in FMS. Qualitative studies may also provide valuable insight into the lived experiences of individuals navigating these intersecting challenges.

REFERENCES

1. Cooksey R, Choy E. Exploring gender differences, medical history, and treatments used in patients with fibromyalgia in the UK using primary-care data: a retrospective, population-based, cohort study. *Lancet Rheumatol* 2022; 4: S20.
2. Casale R, Atzeni F, Bazzichi L, et al. Pain in Women: A Perspective Review on a Relevant Clinical Issue that Deserves Prioritization. *Pain Ther* 2021; 10: 287–314.
3. eClinicalMedicine. Gendered pain: a call for recognition and health equity. *eClinicalMedicine* 2024; 69: 102558.
4. Nurofen. *Gender Pain Gap Index Report Year 3*, https://www.nurofen.co.uk/static/nurofen-gender-pain-gap-index-report-2024-8eede5529e884b5e496a1c02beea4210.pdf (2024, accessed 15 July 2025).
5. Hintz EA. “It’s All in Your Head”: A Meta-Synthesis of Qualitative Research About Disenfranchising Talk Experienced by Female Patients with Chronic Overlapping Pain Conditions. *Health Commun* 2023; 38: 2501–2515.
6. Guzikevits M, Gordon-Hecker T, Rekhtman D, et al. Sex bias in pain management decisions. *Proc Natl Acad Sci* 2024; 121: e2401331121.
7. Zhang L, Losin EAR, Ashar YK, et al. Gender Biases in Estimation of Others’ Pain. *J Pain* 2021; 22: 1048–1059.
8. Coughlin SS, Vernon M, Hatzigeorgiou C, et al. Health Literacy, Social Determinants of Health, and Disease Prevention and Control. *J Environ Health Sci* 2020; 6: 3061.

9. Fentazi D, Pester BD, Yamin JB, et al. Why is low educational attainment linked to worse pain and function in fibromyalgia? *J Pain* 2025; 27: 104764.
10. Zajacova A, Lawrence EM. The relationship between education and health: reducing disparities through a contextual approach. *Annu Rev Public Health* 2018; 39: 273–289.

Table S1. Results of Wilcoxon Test for Differences Between Sexes

Variable	N	Distribution[a]	W Statistic	Z Value	*p*	*r*
Frequency of FMS flares (weeks)	324	Not Similar	40581	3.85	<.001**	0.21
Duration of FMS flares (days)	309	Similar	39331	7.63	<.001**	0.43
Severity of Chronic Widespread Pain	379	Not Similar	60442	6.53	<.001**	0.34
Severity of Fatigue	376	Similar	61321.50	8.14	<.001**	0.42
Number of Symptoms	383	Not Similar	67166	12.74	<.001**	0.65
Number of Additional Diagnoses	383	Not Similar	60069.50	5.15	<.001**	0.26
Months Taken to Receive Diagnosis*	319	Not Similar	44174	10.38	<.001**	0.58
Number of MDs seen before Diagnosis	317	Similar	38960	4.05	<.001**	0.23
Number of Current MDs Treating Symptoms	383	Similar	56958.50	2.33	.020*	0.12
Number of Current Treatments	383	Not Similar	65446.50	10.98	<.001**	0.56
Difficulty of Receiving a Diagnosis	378	Not Similar	62039.50	9.17	<.001**	0.47
Difficulty of Finding a Specialist	376	Not Similar	62087.50	10.03	<.001**	0.52
Perceived Understanding of FMS	361	Not Similar	54246	5.72	<.001**	0.30
Perceived Understanding of FMS Causes	366	Not Similar	54031.50	4.44	<.001**	0.23
Perceived Understanding of FMS from Family	351	Not Similar	43879.50	-4.21	<.001**	-0.22
Perceived Understanding of FMS from MDs	355	Similar	43128	-5.39	<.001**	-0.29
Perceived Level of Caring from MDs	363	Not Similar	50403	0.42	.67	0.02
Perceived Level of Negative Judgment from MDs	358	Not Similar	49311	1.91	.056	0.10
N Total Sources Sought	383	Not Similar	62798.50	8.16	<.001**	0.42
N Social Media Sites Sought	383	Not Similar	55496	0.01	.99	0.00
N Health Websites Sought	383	Not Similar	60696	5.95	<.001**	0.30

*Note. *p<.05; **p<.001*

a=distribution of scores across group assessed visually through boxplots.

Table S2. Results of Kruskal-Wallis H Test for Differences Between Educational Attainment Groups

Variable	N	Distribution[a]	H Statistic	DF	*p*
Frequency of FMS flares (weeks)	325	Not Similar	7.90	4	.095
Duration of FMS flares (days)	310	Similar	13.91	4	.008*
Severity of Chronic Widespread Pain	380	Not Similar	36.77	4	<.001**
Severity of Fatigue	377	Not Similar	33.74	4	<.001**
Number of Symptoms	384	Not Similar	37.46	4	<.001**
Number of Additional Diagnoses	384	Not Similar	9.80	4	.044*
Months Taken to Receive Diagnosis*	320	Not Similar	17.03	4	.002*
Number of MDs seen before Diagnosis	317	Similar	3.68	4	.451
Number of Current MDs Treating Symptoms	384	Similar	1.48	4	.831
Number of Current Treatments	384	Similar	13.66	4	.008*
Difficulty of Receiving a Diagnosis	379	Not Similar	13.68	4	.008*
Difficulty of Finding a Specialist	377	Not Similar	18.88	4	<.001**
Perceived Understanding of FMS	362	Not Similar	3.45	4	.486
Perceived Understanding of FMS Causes	367	Not Similar	13.44	4	.009*
Perceived Understanding of FMS from Family	352	Not Similar	2.42	4	.659
Perceived Understanding of FMS from MDs	356	Similar	7.00	4	.136
Perceived Level of Caring from MDs	364	Similar	3.21	4	.524
Perceived Level of Negative Judgment from MDs	359	Not Similar	1.01	4	.908
N Total Sources Sought	384	Similar	7.18	4	.127
N Social Media Sites Sought	384	Not Similar	3.80	4	.434
N Health Websites Sought	384	Similar	4.05	4	.399

*Note. DF= degrees of freedom; *p<.05; **p<.001*

a=distribution of scores across group assessed visually through boxplots.

Table S4. Results of Kruskal-Wallis H Test for Differences Between Employment Groups

**Variable**	**N**	**Distribution**[a]	**H Statistic**	**DF**	***p***
Frequency of FMS flares (weeks)	325	Not Similar	14.80	3	.002*
Duration of FMS flares (days)	310	Similar	44.68	3	<.001**
Severity of Chronic Widespread Pain	380	Not Similar	73.01	3	<.001**
Severity of Fatigue	377	Similar	69.17	3	<.001**

Table S3. Medians and Interquartile Ranges Across Educational Attainment Groups

Variable	No Qualifications		GCSE		A-Level		UG Degrees		PG Degrees	
	Md	IQR	Md	IQR	Md	IQR	Md	IQR	Md	IQR
Frequency of FMS flares (weeks)	1.5	1.5	2.5	2.25	2	1.13	2	2	3.25	5.25
Duration of FMS flares (days)	3.5	2	4.5	4	3	3	3	2.63	6.5	8.5
Severity of Chronic Widespread Pain	8	1.25	7	3	7	3	6	3	6	3.25
Severity of Fatigue	9	1	8	3	7.5	3	6	3	8	4
Number of Symptoms	13	1.25	12	2	11	12	7	11	12	4
Number of Additional Diagnoses	24	54.75	21	44.75	8	29.5	1	20.75	15	71.5
Months Taken to Receive Diagnosis	3	4	3	3	2	2	2	1	2	2
Number of MDs seen before Diagnosis	2	1.75	2	1.88	2	1.25	2	1	3	3
Number of Current MDs Treating Symptoms	1	0	1	0	1	0	1	0	1	0
Number of Current Treatments	3	2	3	2	3	3	1	2	3	2
Difficulty of Receiving a Diagnosis	4	1	4	3	3	3	3	2.5	4	3
Difficulty of Finding a Specialist	5	1	4	2	3	2	3	3	4	2
Perceived Understanding of FMS	4	2.5	4	1	4	1	3	1	4	1
Perceived Understanding of FMS Causes	4	2.5	3	1	3	1	3	1.75	3	2
Perceived Understanding of FMS from Family	3	1	3	2	3	2	3	2	3	2
Perceived Understanding of FMS from MDs	2.5	1.75	3	2	3	2	3	2	3	2
Perceived Level of Caring from MDs	2	1.75	3	2	3	2	3	2	3	2
Perceived Level of Negative Judgment from MDs	3	2	3	2	3	2	3	1.25	3	2
N Total Sources Sought	2	2.5	2	2	2	2	1	2	3	2
N Social Media Sites Sought	1	1.25	1	0	1	1	1	0	1	1
N Health Websites Sought	1.5	3	1	2	1	2	1	2	2	2

Note. *UG= UG; PG= PG; Md= Median; IQR= Interquartile Range;*

Table S5. Medians and Interquartile Ranges Across Employment Groups

Number of Symptoms	384	Not Similar	133.24	3	<.001**
Number of Additional Diagnoses	384	Not Similar	12.48	3	.006*
Months to Diagnosis	320	Not Similar	79.42	3	<.001**
Number of MDs seen before Diagnosis	317	Similar	6.95	3	.074
Number of Current MDs Treating Symptoms	384	Similar	8.43	3	.038*
Number of Current Treatments	384	Similar	65.91	3	<.001**
Difficulty of Receiving a Diagnosis	379	Not Similar	45.73	3	<.001**
Difficulty of Finding a Specialist	377	Similar	52.51	3	<.001**
Perceived Understanding of FMS	362	Similar	28.89	3	<.001**
Perceived Understanding of FMS Causes	367	Not Similar	26.50	3	<.001**
Perceived Understanding of FMS from Family	352	Similar	9.89	3	.019*
Perceived Understanding of FMS from MDs	356	Not Similar	26.53	3	<.001**
Perceived Level of Caring from MDs	364	Not Similar	8.33	3	.040*
Perceived Level of Negative Judgment from MDs	359	Not Similar	16.30	3	.001**
N Total Sources Sought	384	Not Similar	41.10	3	<.001**
N Social Media Sites Sought	384	Not Similar	1.03	3	.794
N Health Websites Sought	384	Not Similar	21.11	3	<.001**

*Note. DF= degrees of freedom; *p<.05*
a=distribution of scores across group assessed visually through boxplots.

Variable	Unemployed		Part-Time Employed		Full-Time Employed		Self-Employed	
	Md	IQR	Md	IQR	Md	IQR	Md	IQR
Frequency of FMS flares (weeks)	3	3	3	3	2	1	3	1.38
Duration of FMS flares (days)	5	5.5	4	4	3	2	5	7
Severity of Chronic Widespread Pain	8	2	7	3	5	3	6	3.25
Severity of Fatigue	8	3	8	3	6	4	8	3.25
Number of Symptoms	12	2	11	3	1	9	13	3
Number of Additional Diagnoses	3	3	2	2	2	1	2.5	2.5
Months Taken to Receive Diagnosis*	24	39	12	21	0.5	3.75	24	30
Number of MDs seen before Diagnosis	3	2	2.5	2	2	1	2	1.5
Number of Current MDs Treating Symptoms	1	0	1	0	1	0	1	0
Number of Current Treatments	3	2	3	2	1	2	4	2.25
Difficulty of Receiving a Diagnosis	4	2	4	3	2	3	4	3
Difficulty of Finding a Specialist	4	2	4	2	2	2	4	2
Perceived Understanding of FMS	4	1	4	1	3	1	4	1.25
Perceived Understanding of FMS Causes	3	1	3	1	3	1	3.5	2
Perceived Understanding of FMS from Family	3	2	3	1	3	2	3	3
Perceived Understanding of FMS from MDs	3	2	3	2	4	3	3	2
Perceived Level of Caring from MDs	2	2	3	2	3	1	4	3
Perceived Level of Negative Judgment from MDs	3	1	2	1	3	1	3	3
N Total Sources Sought	2	2	2	2	1	1	3	2
N Social Media Sites Sought	1	1	1	1	1	0	1	0.25
N Health Websites Sought	2	2	2	2	1	0	2.5	3.25

Note. Md= Median; IQR= Interquartile range.
